# Clinical validation of the nursing diagnostic proposition perioperative thirst*

**DOI:** 10.1590/1518-8345.6621.3975

**Published:** 2023-08-04

**Authors:** Leonel Alves do Nascimento, Marilia Ferrari Conchon, Aline Korki Arrabal Garcia, Marcos Venícios de Oliveira Lopes, Lígia Fahl Fonseca

**Affiliations:** 1 Hospital Doutor Anísio Figueiredo, Unidade Cirúrgica, Londrina, PR, Brasil.; 2 Universidade Estadual de Londrina, Departamento de Enfermagem, Londrina, PR, Brasil.; 3 Universidade Federal do Ceará, Departamento de Enfermagem, Fortaleza, CE, Brasil.

**Keywords:** Thirst, Perioperative Nursing, Clinical Decision-Making, Nursing Methodology Research, Nursing Diagnosis, Evidence-Based Nursing, Sed, Enfermería Perioperatoria, Toma de Decisiones Clínicas, Investigación Metodológica en Enfermería, Diagnóstico de Enfermería, Enfermería Basada en la Evidencia, Sede, Enfermagem Perioperatória, Tomada de Decisão Clínica, Pesquisa Metodológica em Enfermagem, Diagnóstico de Enfermagem, Enfermagem Baseada em Evidências

## Abstract

**Objective:**

to verify the clinical validity of the proposition of a new nursing diagnosis called perioperative thirst, based on the diagnostic accuracy of its clinical indicators, including the magnitude of effect of its etiological factors.

**Method:**

clinical diagnostic validation study with a total of 150 surgical patients at a university hospital. Sociodemographic variables and clinical indicators related to thirst were collected. The latent class analysis technique was used.

**Results:**

two models of latent classes were proposed for the defining characteristics. The model adjusted preoperatively included: dry lips, thick saliva, thick tongue, desire to drink water, caregiver report, dry throat and constant swallowing of saliva. In the postoperative period: dry throat, thick saliva, thick tongue, constant swallowing of saliva, desire to drink water, bad taste in the mouth. The factors related to “high ambient temperature” and “dry mouth” are associated with the presence of thirst, as well as the associated conditions “use of anticholinergics” and “intubation”. The prevalence of thirst was 62.6% in the pre and 50.2% in the immediate postoperative period.

**Conclusion:**

the diagnostic proposition of perioperative thirst showed good accuracy parameters for its clinical indicators and etiological effects. This proposition in a nursing taxonomy will allow greater visibility, appreciation and treatment of this symptom.

## Abstract (es)

**Objetivo:**

verificar la validez clínica de la proposición de un nuevo diagnóstico de enfermería denominado sed perioperatoria, basado en la precisión diagnóstica de sus indicadores clínicos, incluyendo la magnitud del efecto de sus factores etiológicos.

**Método:**

estudio de validación clínica diagnóstica con 150 pacientes quirúrgicos en un hospital universitario. Se recogieron variables sociodemográficas e indicadores clínicos relacionados con la sed. Se utilizó la técnica de análisis de clases latentes.

**Resultados:**

se propusieron dos modelos de clases latentes para las características definitorias. El modelo ajustado en el preoperatorio incluía: labios resecos, saliva espesa, lengua espesa, ganas de beber agua, informe del cuidador, garganta seca y deglución constante de saliva. En el postoperatorio: sequedad de garganta, saliva espesa, lengua espesa, constante deglución de saliva, ganas de beber agua, mal gusto en la boca. Los factores relacionados “temperatura ambiente elevada” y “sequedad de boca” se asocian a la presencia de sed, así como las condiciones asociadas “uso de anticolinérgicos” e “intubación”. La prevalencia de sed fue del 62,6% en el preoperatorio y del 50,2% en el postoperatorio inmediato.

**Conclusión:**

la proposición diagnóstica de la sed perioperatoria mostró buenos parámetros de precisión de sus indicadores clínicos y efectos etiológicos. Esta propuesta en una taxonomía de enfermería permitirá una mayor visibilidad, apreciación y tratamiento de este síntoma.

## Abstract (pt)

**Objetivo:**

verificar a validade clínica da proposição de um novo diagnóstico de enfermagem denominado sede perioperatória, com base na acurácia diagnóstica de seus indicadores clínicos, incluindo a magnitude de efeito de seus fatores etiológicos.

**Método:**

estudo de validação clínica diagnóstica com 150 pacientes cirúrgicos em um hospital universitário. Foram coletadas variáveis sociodemográficas e indicadores clínicos relacionados à sede. Empregou-se a técnica de análise de classe latente.

**Resultados:**

dois modelos de classes latentes foram propostos para as características definidoras. O modelo ajustado no pré-operatório incluiu: lábios ressecados, saliva grossa, língua grossa, vontade de beber água, relato do cuidador, garganta seca e constante deglutição de saliva. No pós-operatório: garganta seca, saliva grossa, língua grossa, constante deglutição de saliva, vontade de beber água, gosto ruim na boca. Os fatores relacionados Temperatura do ambiente elevada e Boca seca estão associados à presença de sede, assim como as condições associadas Utilização de anticolinérgicos e Intubação. A prevalência de sede foi de 62,6% no pré-operatório e 50,2% no pós-operatório imediato.

**Conclusão:**

a proposição diagnóstica de sede perioperatória apresentou bons parâmetros de acurácia de seus indicadores clínicos e efeitos etiológicos. Essa proposição em uma taxonomia de enfermagem permitirá maior visibilidade, valorização e tratamento desse sintoma.

**Highlights:**

**(1)** Evaluates the accuracy of the proposition of the nursing diagnosis perioperative thirst;

**(2)** Allows refined diagnosis for use in clinical practice, teaching and research;

**(3)** Strengthens the systematization of perioperative nursing care;

**(4)** Highlights thirst management as part of care, considering its high prevalence and discomfort;

**(5)** Presents a structure with good accuracy parameters which are representative of thirst.

## Introduction

Perioperative thirst is a sensory, physiological and subjective experience that refers to the desire to drink water in order to restore body fluid homeostasis, generating intense discomfort when not attended to^(^ 1 ^-^ 5 ^)^.

In a scenario where the prevalence of thirst is high, ranging from 79.5%^(^ 6 ^)^ to 89.8%^(^ 7 ^)^ in adults, reaching more than 80% in pediatric patients at different levels of intensity^(^ 8 ^)^, the intentional inclusion of this discomfort in nursing monitoring becomes relevant and urgent^(^ 2 ^,^ 7 ^,^ 9 ^)^.

Thirst negatively influences the surgical experience and its presence is pressing and imperative. It is one of the main discomforts mentioned in this period, both in the current experience and in the memory of discomforts from previous surgeries, and it can be even worse than pain^(^ 10 ^-^ 12 ^)^. Despite this, many professionals believe that thirst is a consequence of the surgical procedure and must be tolerated by the patient. Therefore, taking care of thirst is still undervalued, underassessed, undermeasured and undertreated by the nursing team^(^ 7 ^,^ 10 ^,^ 13 ^)^.

Other discomforts and complications relevant to the surgical patient are widely researched and included in clinical protocols, directing their care. However, the clinical relevance of thirst is often minimized when compared to other discomforts and its management is still not carried out in a systematic way^(^ 2 ^,^ 7 ^,^ 10 ^)^.

Considering this scenario, Brazilian researchers developed a Thirst Management Model, which is composed of four pillars: Identification, Measurement, Safety Assessment and Administration of a thirst relief strategy^(^ 2 ^)^. This model, when used with the surgical patient in the pre and immediate postoperative period (IPP), allows the team to carry out adequate, safe, and effective management of this discomfort.

By including thirst management in nursing care, it is expected that the patient can have his thirst diagnosed and relief measures applied in a systematic and standardized way. This care allows the patient to have a surgical experience with the least possible suffering, optimizing his recovery and actively improving the quality of care provided^(^ 2 ^,^ 7 ^,^ 10 ^)^.

One way to emphasize its importance would be to incorporate thirst in international classifications and terminologies, highlighting its main signs and symptoms, as well as its possible causal factors. In this context, the NANDA International taxonomy of nursing diagnoses (NANDA-I) presents an organization of diagnoses that incorporates these elements and has been used in several countries, in addition to serving as a starting point for the development of other nursing terminologies and classifications^(^ 14 ^)^. Historically, the development of a new diagnosis follows concept and content analysis, culminating in clinical validation.

It is in this context that the proposition of the nursing diagnosis (ND) for perioperative thirst is based. In the daily care provided by the nursing team, thirst is frequently observed and must have its standardized management. Including perioperative thirst in the nursing diagnostic taxonomy will allow the surgical patient’s thirst to be identified and treated.

This proposition has already been evaluated in relation to the concept analysis^(^ 3 ^)^ and its content^(^ 4 ^)^, with the definition: “Sensory, physiological and subjective experience that refers to the desire to drink water in order to restore the homeostasis of body fluids, generating intense discomfort when not attended to”. Homeostatic and non-homeostatic mechanisms participate in the genesis and satiety of thirst and resulting in a diagnostic framework composed of nine defining characteristics: dry mouth, dry throat, dry lips, thick saliva, thick tongue, constant swallowing of saliva, desire to drink water, bad taste in the mouth, caregiver report. The resulting related factors (RF) were: pre- and postoperative fasting, mouth breathing, dehydration, hypovolemia, insensible loss of hydration through breathing, dry mouth, habit of drinking water, high ambient temperature. The associated conditions (AC) defined after the analysis were: intubation, use of anticholinergics, fluid restriction.

This study aimed to verify the clinical validity of the proposition of a new nursing diagnosis called perioperative thirst, based on the diagnostic accuracy of its clinical indicators, including the magnitude of the effect of its etiological factors.

## Method

### Study design

Clinical validation study of the proposition of the ND “Perioperative Thirst” in two moments (pre- and immediate postoperative period).

### Setting and period

The study was carried out in the Post Anesthesia Care Unit (PACU) of the University Hospital of the State University of Londrina, in the city of Londrina, Paraná, Brazil. It is a large hospital, with 419 clinical and surgical beds. The surgical center has seven operating rooms and six beds for anesthetic recovery, performing an average of 700 surgeries *per* month, serving different specialties and surgical sizes. Patients stay in a preoperative room until the moment of the surgical procedure. After the end of the surgery, they are sent to the PACU, where they are monitored until they reach the safety criteria for discharge and transference to the hospitalization units. The ratio of nurses *per* patient is 1:6. Surgical specialties included gynecological, orthopedic, urological and ophthalmological surgical. The procedures could be elective or urgent/emergency. Data collection was carried out during December 2019 to January 2020.

### Population and selection criteria

The study population consisted of patients who underwent elective and urgent surgical procedures. As inclusion criteria, were stipulated patients aged at least 12 years old and who, when asked about their orientation in time and space, responded assertively. As exclusion criteria, those who were unable to respond to the guidance questions in the IPP and those who were transferred while still on mechanical ventilation to the Intensive Care Unit (ICU).

### Sample definition

For the sample calculation, the proposed recommendations were observed for carrying out diagnostic accuracy studies through latent class analysis, in which 5 to 30 patients are indicated for each clinical indicator^(^ 15 ^)^. In this study, 16 patients were chosen for each defining characteristic (DC)^(^ 4 ^)^, resulting in 150 patients. Consecutive convenience sampling was used, in which patients were included as they were admitted to the operating room.

### Study variables

The sociodemographic variables were: age, gender, skin color, education, weight, height. Variables related to the surgical procedure and site were considered: surgical specialty, scheduling modality, physical status assessment by the American Society of Anesthesiologists, fasting time, bleeding reported in the operative description by the surgeon and anesthesiologist, measurement of thirst intensity by verbal scale numeric (zero, no thirst, to ten, worst possible thirst) and thirst discomfort using the Perioperative Thirst Discomfort Scale (EDESP), an instrument constructed and validated for the Brazilian population, which assesses seven items related to thirst, resulting in an intensity score from zero to 14^(^ 16 ^)^ and measurement of thirst intensity.

The variables referring to the ND components were evaluated and recorded as absent or present. The operational definitions^(^ 3 ^-^ 4 ^)^ can be seen in Figure 1.

**Figure 1 -:** Components of the Perioperative Thirst Nursing Diagnosis proposal and its operational definitions

Defining Characteristic	Operational Definition
Dry mouth	The patient is asked to open the mouth and stick out the tongue. Evidence of any apparent dryness, such as dry, sticky mucous membrane, frothy or sticky saliva, or no visible saliva, whitish appearance, or bleeding spots is evaluated. Complement your assessment by questioning the patients about their current perception of dryness in the mouth, comparing it with a condition in which he considers normal moisture in the mouth. *Conclusion*: If one or two evaluated conditions were met, the patient has a dry mouth.
Dry throat	Ask the patient to open his mouth. Assess the absence of moisture in the distal portion of the mouth with the aid of a flashlight. Complement your assessment by questioning the patients about their current perception of throat dryness, burning sensation, throat discomfort such as “scratching”, compared to a condition in which he considers throat moisture normal. *Conclusion*: If the patients have one or more of the evaluated characteristics, they have a dry throat.
Dry lips	Inspect the lips, assessing the presence of cracks, scaling, inflammation, bleeding, whitish or reddish coloration, and decreased mobility. Observe if during the evaluation the patient uses the tongue to moisten the lips. Complement your assessment by asking the patients if they feel their lips are dry compared to a condition where the lips are normal. *Conclusion*: If the patients have one or more of the evaluated characteristics, they have dry lips.
Thick saliva	Inspect the patient’s oral cavity. Observe whether the saliva has a thick appearance, with little fluidity. Complement your assessment by asking the patients if they feel that their saliva is thick, compared to a condition in which their saliva is normal. *Conclusion*: If the patients have one or more of the evaluated characteristics, they have thick saliva.
Thick tongue	Inspect the appearance of the patient’s tongue. Note if there is visible enlargement of the tongue, erythema, fissures. Complement your assessment by asking the patients if they feel that their tongue is thick compared to a condition where their tongue is normal. *Conclusion*: If the patients have one or more of the evaluated characteristics, they have a thick tongue.
Defining Characteristic	Operational Definition
Constant swallowing of saliva	Observe the swallowing movements. Actions of constant movement of the tongue in search of saliva, followed by an attempt to swallow. Supplement your assessment by asking the patients if they are trying to swallow saliva to moisten their throat. *Conclusion*: If the patients have one or more of the evaluated characteristics, they have constant swallowing of saliva.
Desire to drink water	Patient verbalizes the desire to drink water. Ask the patient if he feels like drinking water. *Conclusion*: If the patient responds affirmatively, consider this feature to be present.
Bad taste in the mouth	Ask the patient if he has a bad taste in his mouth. *Conclusion*: If the patient responds affirmatively, consider this feature to be present.
Caregiver’s report	The caregiver reports that the patient has signs that indicate that he is thirsty. Ask the caregiver or family member about the presence of signs that indicate that the patient is thirsty. *Conclusion*: If the caregiver answers in the affirmative, consider this characteristic to be present. Additionally, write down what characteristics he observed.
Related Factor	Operational Definition
Pre- and post-operative fasting	Evaluate the time the patient has been without ingesting solid foods and liquids. Ask the patient when his last intake of solid food and liquids was.
Oral breathing	Ask the patient to take a deep breath and watch for mouth breathing. He may have his mouth slightly open while breathing. Check for nasal obstruction.
Dehydration	Note the presence of dry mucous membranes, decreased salivary flow, decreased skin turgor, decreased urinary frequency. - *Dry mucous membranes*: The patient is asked to open his mouth and stick out his tongue. Evidence of any apparent dryness, such as dry, sticky mucosa, frothy or sticky saliva, or absence of visible saliva, is evaluated. To complement the assessment, the patient should be asked about his current perception of dryness in the mouth, comparing it with a condition in which he considers that the moisture in the mouth is normal. *- Skin turgor:* Make a fold on the back of the patient’s hand and observe the formation and maintenance of a skin fold. If the fold continues after the evaluator’s hand is removed, it is deduced that the skin turgor is reduced*.* *- Evaluate fasting time* *- Water balance, if any* *- Assess blood loss* *- Water replacement* *In children* Observe signs of dehydration in the child, such as: fatigue, dry mucous membranes, dry lips, return of the abdominal skinfold for more than 2 seconds, crying without tears, irritability, sunken eyes. Note fontanelles. Ask the caregiver about changes in the child related to dehydration (skin turgor, fontanelles, general aspects).
Insensible loss of moisture from respiration	*In the postoperative period:* Evaluate the inhalational anesthetic procedure and the use of oxygen supplementation by nasal catheter or mask without humidification.
Hypovolemia	Evaluate the description of bleeding during the surgical procedure or in the postoperative period. Check for hypotension, sweating, tachycardia, and other signs of shock. *Anesthetic procedure*: Use of spinal anesthesia results in vasodilation caused by sympathetic blockade. Agents employed in general anesthesia initially decrease the body’s ability to perform vasoconstriction. Write down which anesthetic procedure was used. *Hypotension:* Check blood pressure. Compare the current pressure with the preoperative baseline pressure: if it is 20% lower than the baseline pressure, the patient can be considered hypotensive. *Heart rate:* Check heart rate by palpating and counting the radial pulse. The patient will be tachycardic if the observed value is equal to or greater than 100 beats *per* minute. *Blood loss:* Check the documents that report the intraoperative period for the description of the bleeding: small, medium or large? Check the appearance of the dressing. *Sweating:* Check for sweating.
Dry mouth	Evaluation of the oral cavity. The patient is asked to open the mouth and stick out the tongue. Evidence of any apparent dryness, such as dry, sticky mucous membrane, frothy or sticky saliva, or no visible saliva, whitish appearance, or bleeding spots is evaluated. Complement your evaluation by questioning the patient about his current perception of dryness in the mouth, comparing it with a condition in which he considers normal moisture in the mouth. *Conclusion*: If one or two evaluated conditions were met, the patient has a dry mouth.
Habit of drinking water	Ask the patient how many times a day they usually drink fluids and in what quantity, supplementing with the information below: *- Frequency of consumption:* *- Consumption/beverage preference:* *- Period of the day of greatest consumption:* *- Temperature preference:*
High ambient temperature	Measure the ambient temperature. Ask if he’s hot.
Associated Conditions	Operational Definition
Intubation	Assess whether there was endotracheal intubation during the anesthetic procedure. Was there orotracheal intubation? What is the duration of the anesthetic procedure? *- Beginning of the anesthetic procedure:* *- End of the anesthetic procedure:*
Water restriction	Evaluate the existence of restriction of intake or infusion of liquids according to the patient’s report or in the medical record. Evaluate in the medical record or the patient’s report the existence of restriction of fluid intake or infusion.
Use of muscarinic and nicotinic anticholinergics	Assess whether anticholinergics were used during the anesthetic-surgical procedure. *Anticholinergic drugs*: atropine, biperiden, benactizine, buscopan, dicyclomine, homatropine, ipratropium, trihexaphenid, tropicamide

### Study procedure

The data collection tool was composed of three parts. First, social and demographic characterization data, followed by clinical and anesthetic-surgical procedure data, and third, information on the ND components. The data collection tool was submitted to a pre-test, with 10% of the intended sample for the study, evaluating whether the instrument presented a logical sequence of application, completion and whether all items were feasible to answer. The pre-test did not result in any significant changes and was included in the final sample. Data collection was carried out by the main researcher, a nurse and two scientific initiation students, all members of the Perioperative Thirsty Study and Research Group. Training was carried out with a workload of four hours by the main researcher, containing information on the phenomenon of perioperative thirst, its related factors, associated conditions, defining characteristics, conceptual and operational definitions, as well as research on diagnostic accuracy. After this training, all those responsible for data collection used the collection instrument and, together with the main researcher, performed ten assessments before starting the individual collection. These procedures allowed the calibration of data collection and the reduction of doubts related to this step.

Data collection was performed on the same patient at two different times: first, before the surgical procedure, in the preoperative room and, upon agreeing to participate in the research, they signed the consent form; in the second moment, in the immediate postoperative period, in the Post Anesthesia Care Unit. Patients under 18 years old and their legal guardians were invited to participate, and if they agreed, they signed the assent and consent forms. At both moments of data collection, the patient was evaluated regarding his level of orientation in time and space.

In the IPP, if the patient was unconscious, drowsy or disoriented, the assessment was postponed for the next 15 minutes, until the orientation conditions were adequate. To assess them, the following questions were asked based on the Orientation item of the Montreal Cognitive Assessment (MoCA)^(^ 17 ^)^: What is your name? Where are you at this moment? What day is today? Which surgery did you perform? After data collection, management of the patient’s thirst was carried out according to the practice of the institution^(^ 2 ^)^.

### Data analysis

Data were entered into a Microsoft Excel 2016 spreadsheet and analyzed using the statistical software R version 3.6.1. The analysis of latent classes, as well as the presentation of accuracy measures such as sensitivity and specificity^(^ 15 ^)^, were carried out with the aid of the “poLCA” and “randomLCA”^(^ 18 ^)^ packages.

The descriptive analysis of categorical data was calculated in absolute frequencies absolute frequencies, percentages and their 95% confidence intervals (CI). For quantitative variables, measures of central tendency and dispersion were used. Verification of adherence to the normal distribution was carried out using the Shapiro-Wilk test.

To estimate the best adjusted model, the principle of parsimony was used, which defines that the best model is the one that presents a better fit with a smaller number of parameters to be estimated. A model of two latent classes was developed, allowing the identification of the prevalence of the diagnosis, as well as the accuracy measures, with the respective 95% CI.

Two latent class models were proposed, the first referring to DC in the preoperative period and the second one, in the IPP. The models were adjusted for all variables and the likelihood ratio test (G²) was applied to verify the quality of the fit. In cases where a good fit was not presented, DC with sensitivity and specificity lower than 50% were sequentially removed.

To define the prevalence of thirst, a table of post-test probabilities calculated for all combinations of indicators included in the final model in the pre and IPP was used, resulting in diagnostic inference for all patients.

The chi-square test was used, as well as the odds ratio and its confidence intervals (95%) using Fisher’s test. For quantitative variables, normality was verified for each subgroup using the Shapiro-Wilk test. Upon presentation of adherence to the normal distribution, the homogeneity test was performed (Bartlett’s test) and thus the t test (equal or unequal variances). For variables that did not adhere to normal distribution, the Mann-Whitney test was used.

### Ethical aspects

The research project received a favorable opinion from the Research Ethics Committee of the State University of Londrina – 94634018.5.0000.5231. All recommendations of the National Research Ethics Committee (CONEP) were observed.

## Results

A total of 160 patients were approached, but ten patients were not evaluated postoperatively, as they were referred to the ICU directly from the operating room. A total of 150 patients participated in the study, predominantly female, self-declared white, with a low level of education, since most reported having elementary school, although half of the participants were young adults aged 42 years or less.

The mean preoperative fasting time was high (13:10 hours), despite the predominance of elective surgeries, which would provide a shorter preoperative fasting time (Table 1). The surgical clinics surveyed were: gynecological (26%), orthopedic (20%), urological (18.7%), ophthalmological (12.7%), digestive system (6%), otorhinolaryngological (5.3%), oral and maxillofacial (3.3%), vascular (2%), general surgery (1.3%), head and neck (1.3%), plastic surgery (1.3%), cardiology (0.7%), thoracic (0.7%) and neurological (0.7%).

**Table 1 -:** Distribution of sociodemographic and clinical characteristics of study participants (150 participants). Londrina, PR, Brazil, 2020

Variables			n*		%^†^		95%CI^‡^		
**Gender**									
Female			89		59.3		51.0 – 67.1		
Male			61		40.7		32.8 – 48.9		
**Color or race**									
White			84		56.0		47.6 – 64.0		
Brown			45		30.0		22.9 – 38.1		
Black			20		13.3		08.5 – 20.0		
Yellow			01		00.7		0.00 – 04.2		
**Education**									
No schooling			05		03.3		01.2 – 08.0		
Elementary School			71		47.3		39.1 – 55.6		
High school			54		36.0		28.4 – 44.2		
University education			17		11.3		06.9 – 17.7		
Did not answer			03		02.0		0.5 – 06.1		
**Scheduling modality**									
Elective			98		65.3		57.0 – 72.7		
Urgency			52		34.7		27.2 – 42.9		
**Classification**									
ASA^§^ 1			55		36.7		29.0 – 44.9		
ASA^§^ 2			88		58.7		50.3 – 66.5		
ASA^§^ 3			05		03.3		01.2 – 08.0		
Absent			02		01.3		0.2 – 05.2		
**Intraoperative bleeding**									
Small			123		82.0		74.7 – 87.6		
Average			23		15.3		10.1 – 22.3		
Big			03		02.0		0.5 – 06.1		
Absent			1		0.7		0.00 – 04.2		
Variables	Mean	Standard Derivation		Median		IIQ^||^		P-value^¶^	
Age	43.1	18.5		41.5		33.00		<0.001	
Weight (Kg)	77.36	18.85		75.00		21		0.001	
Height (Centimeters)	164.7	8.83		165.00		14		0.006	
Fasting time (Hours)	13:10	5:11		12:50		5:52		0.000	
Intensity of thirst	4.62	3.6		5		8		0.000	
Thirst discomfort	4.88	3.9		4		14		0.000	

*n = Absolute Frequency; ^†^% = Percentage; ^‡^CI 95% = Confidence Interval of 95%; ^§^ASA = American Society of Anesthesiologists; ^||^IIQ = Interquartile Range; ^¶^p-value = Shapiro-Wilk

Five of the nine DCs had frequencies of occurrences greater than 50% in the preoperative period and seven in the IPP. It is observed that with the exception of the DC Caregiver’s report, all the DCs had a higher frequency of occurrence in the IPP, when compared to the preoperative period. The DC Dry mouth, Dry throat and Dry lips were more frequent in the IPP. All patients presented the pre- and postoperative fasting RF; the RF Dehydration, Insensible loss of hydration through breathing and Hypovolemia were only found in patients in the IPP, similarly to CA Intubation and Use of anticholinergics (Table 2).

**Table 2 -:** Distribution of the proposition components of the nursing diagnosis Perioperative Thirst in the pre and postoperative period, according to the study population (150 participants). Londrina, PR, Brazil, 2020

Defining Characteristics	Preoperative			Immediate Postoperative		
	n*	%^†^	95%CI^‡^	n*	%^†^	95%CI^‡^
Dry mouth	113	75.3	67.5 – 81.8	130	86.7	79.9 – 91.4
Dry lips	107	71.3	63.2 – 78.2	124	82.7	75.4 – 88.1
Desire to drink water	96	64.0	55.7 – 71.5	109	72.7	64.6 – 79.4
Constant swallowing of saliva	94	62.7	54.3 – 70.3	105	70.0	61.8 – 77.0
Dry throat	87	58.0	49.6 – 65.9	108	72.0	63.9 – 78.8
Thick saliva	73	48.7	40.4 – 56.9	80	53.3	45.0 – 61.4
Thick tongue	69	46.0	37.9 – 54.3	82	54.7	46.3 – 62.7
Bad taste in the mouth	62	41.3	33.4 – 49.6	69	46.0	37.9 – 54.3
Caregiver’s report	01	0.7	0.00 – 04.2	01	0.7	0.00 – 04.2
**Related Factors**	**Preoperative**			**Immediate Postoperative**		
	n*	**%** ^†^	**95%CI** ^‡^	n*	**%** ^†^	**95%CI** ^‡^
Pre- and post-operative fasting	150	100	96.8 – 100.0	150	100	96.8 – 100.0
Habit of drinking water	120	80.0	72.5 – 85.9	120	80.0	72.5 – 85.9
Dry mouth	115	76.7	68.9 – 83.0	128	85.3	78.4 – 90.3
High ambient temperature	28	18.7	12.9 – 26.0	16	10.7	06.4 – 17.0
Oral breathing	04	2.7	0.00 – 07.1	07	4.7	02.0 – 09.7
Dehydration	-	-	-	02	1.3	0.2 – 5.2
Insensible loss of moisture from respiration	-	-	-	29	19.3	13.5 – 26.7
Hypovolemia	-	-	-	03	2.0	0.5 – 06.1
**Associated Conditions**	**Preoperative**			**Immediate Postoperative**		
	n*	**%** ^†^	**95%CI** ^‡^	n*	**%** ^†^	**95%CI** ^‡^
Water restriction	1	0.7	0.00 – 04.2	1	0.7	0.00 – 04.2
Use of anticholinergics	-	-	-	27	18.0	12.3 – 25.2
Intubation	-	-	-	37	24.7	18.1 – 32.4

^*^n = Absolute Frequency; ^†^% = Percentage; ^‡^95%CI = 95% Confidence Interval

Table 3 presents the latent class models of the adjusted defining characteristics for the pre and IPP. Seven DC remained in the model for the preoperative period, with Dry throat and Constant swallowing of saliva presenting better accuracy values. In the IPP, six DC remained, with better accuracy for Bad taste in the mouth. The models showed adequate adjustment according to the likelihood ratio test, demonstrating that there was no difference between the adjusted model and the data from the sample. There was a prevalence of thirst of 62.6% for the pre and 50.2% for the IPP. The DC Dry mouth was not included in any of the models, as it did not have adequate accuracy values.

In the adjusted model for the DCs of the proposition of the ND Perioperative Thirst for the IPP, the DC Bad taste in the mouth showed better accuracy, with high sensitivity and specificity values. The DC Dry throat, Constant swallowing of saliva and Desire to drink water showed better sensitivity values. The best specificity indicators were for the DC Thick saliva and Thick tongue. It is observed that the DC Dry throat and Constant swallowing of saliva again showed high sensitivity values, indicating that these DC are related to the presence of thirst in the patient also in the IPP.

**Table 3 -:** Latent class adjusted model of the defining characteristics found in the pre and immediate postoperative periods (150 participants). Londrina, PR, Brazil, 2020

Preoperative	Sens.^*^	95%CI^†^		Spec.^‡^	95%CI^†^	
Dry lips	0.8918^§^	0.6803	0.9607	0.5848	0.4331	0.7229
Desire to drink water	0.8771^§^	0.7005	0.9473	0.7562	0.4882	0.8961
Constant swallowing of saliva	0.8171^§^	0.7176	0.8860	0.6914^||^	0.5123	0.8223
Dry throat	0.7950^§^	0.6806	0.8722	0.7792^||^	0.5858	0.8935
Thick saliva	0.7005^§^	0.5719	0.7978	0.8706	0.4215	0.9758
Thick tongue	0.6742^§^	0.5476	0.7755	0.8978	0.4981	0.9834
Caregiver’s report	0.0107	0.0003	0.9854	1.0000^||^	0.9996	1.0000
Prevalence: 62.6%	G^2¶^: 75.5		GL^**^:112	p^††^ = 0.997	Entropia:0.77	
**Immediate Postoperative**	**Sens.** ^*^	**95%CI** ^†^		**Spec.** ^‡^	**95%CI** ^†^	
Dry throat	0.9998^§^	0.9730	1.0000	0.5620	0.4317	0.6867
Thick tongue	0.9311	0.2357	0.9956	0.8409^||^	0.5452	0.9476
Thick saliva	0.8820	0.4759	0.9771	0.8144^||^	0.5172	0.9375
Desire to drink water	0.8520^§^	0.7293	0.9236	0.3997	0.3002	0.5217
Constant swallowing of saliva	0.7538^§^	0.6233	0.8364	0.3543	0.2561	0.4631
Bad taste in the mouth	0.6429^§^	0.5153	0.7483	0.7243^||^	0.5925	0.8176
Prevalence: 50.2%	G^2¶^: 59.1		GL^**^: 50	p^††^ = 0.177	Entropy: 0.82	

^*^Sens. **=** Sensitivity; ^†^95%CI= Confidence Interval; ^‡^Spec. = Specificity; ^§^Clinically sensitive indicators; ^||^Clinically specific indicators; ^¶^G2 = Likelihood ratio test; **GL = Degrees of Freedom; ^††^p = Chi-square test

The RF that showed a significant association with the presence of thirst in the preoperative period were high ambient temperature and dry mouth. Having a dry mouth increased the chances of being thirsty by 37.72 and the ambient temperature increased by 3.60 times. No AC showed significance with the presence of thirst in the preoperative period. In the IPP, the RF Dry mouth was associated with the presence of thirst, increasing the chances of being thirsty by 28.20. The AC Use of anticholinergics and Intubation showed a significant association with the presence of thirst, increasing the chances of the patient being thirsty by 2.64 and 4.03, respectively (Table 4).

**Table 4 -:** Association between related factors and conditions associated with the presence and absence of the proposed perioperative nursing diagnosis (150 participants). Londrina, PR, Brazil, 2020

	Preoperative					Immediate Postoperative				
	Perioperative Thirst		Test statistics	Significance	OR (95%CI)*	Perioperative Thirst		Test statistics	Significance	OR (95%CI)*
	Present	Absent				Present	Absent			
**Related Factor: Pre and Postoperative Fasting**										
Present	100	50	-	-	-	77	73	-	-	-
Absent	0	0				0	0			
**Related Factor: Oral breathing**										
Present	3	1	<0.000	1.000^†^	1.51 (0.12-81.13)	4	3	<0,000	1.000^†^	1.28 (0.21-9.02)
Absent	97	49				73	70			
**Related Factor: Dehydration**										
Present	0	0	-	-	-	2	0	0.454	0.497^†^	Inf^‡^ (0.18-Inf^‡^)
Absent	100	50				75	73			
**Related Factor: Insensible loss of moisture from respiration**										
Present	0	0	-	-	-	19	10	2.234	0.135^§^	2.05 (0.83-5.38)
Absent	100	50				58	63			
**Related Factor: High ambient temperature**										
Present	24	4	4.616	0.025^†^	3.60 (1.14-15.19)	12	4	3.025	0,063^†^	3.16 (0.99-14.14)
Absent	76	46				65	69			
**Related Factor: Hypovolemia**										
Present	0	0	-	-	-	3	0	1.254	0.245^†^	Inf^‡^(0.39-Inf^‡^)
Absent	100	50				74	73			
**Related Factor: Dry mouth**										
Present	96	19	59.483	>0.001^†^	37.72 (11.56-164.36)	76	53	19.087	>0,001^†^	28.20 (4.25-1196.76)
Absent	4	31				1	20			
**Related Factor: Habit of drinking water**										
Present	83	37	1.172	0.279^§^	1.709 (0.69-4.19)	64	56	0.602	0,438^§^	1.49 (0.62-3.66)
Absent	17	13				13	17			
**Associated Condition: Water restriction**										
Present	1	0	<0.000	1.000^†^	Inf^‡^ (0.02-Inf^‡^)	1	0	<0,000	1.000^†^	Inf^‡^ (0.02-Inf^‡^)
Absent	99	50				76	73			
**Associated Condition: Use of anticholinergics**										
Present	0	0	-	-	-	19	8	3.892	0.048^§^	2.64 (1.01-7.54)
Absent	100	50				58	65			
**Associated Condition: Intubation**										
Present	0	0	-	-	-	28	9	10.392	0,001^§^	4.03 (1.66-10.63)
Absent	100	50				49	64			

*OR (95%CI) **=** Odds Ratio (95% Confidence Interval); ^†^Fisher’s test; ^‡^Inf = Infinity; ^§^Chi-square test

## Discussion

The uniqueness of this study is evidenced by the analysis of the accuracy of the DC of the proposition of a new Perioperative Thirst ND in patients both in the pre and in the IPP. Evaluating the same patient before and after his surgical procedure allowed a greater understanding of how the resulting attributes behave and the possibility of comparing the presence and absence of these characteristics in the two moments. The results show the development of two models with DCs with high sensitivity and specificity.

Perioperative thirst is an intense and highly prevalent perioperative discomfort^(^ 6 ^-^ 7 ^,^ 9 ^,^ 19 ^-^ 20 ^)^. The literature reveals the recent interest in this topic in the nursing community, with its management being structured in the identification, measurement, safety assessment for carrying out interventions and the application of relief strategies^(^ 2 ^,^ 21 ^)^.

The intensity and discomfort of thirst are high. Studies that assess the intensity of thirst present values similar to or higher than those found in the studied sample: 4.8 (SD 0.7)^(^ 6 ^)^; 6.9 (SD 2.4)^(^ 13 ^)^; 6.9 (SD 2.2)^(^ 20 ^)^; 7.6 (SD 1.6)^(^ 22 ^)^. The use of the EDESP^(^ 16 ^)^ to measure discomfort is still recent, but similar values were also found: 5.0 (SD 3.4)^(^ 6 ^)^; 7.3 (SD 3.7)^(^ 13 ^)^.

Five characteristics had frequencies greater than 50% in the preoperative period, with the DC Dry mouth and Dry lips being the highest incidences. In the IPP, seven of the nine DCs had an incidence greater than 50%, again highlighting the DC Dry mouth and Dry lips with the highest occurrences. The study of the characteristics related to perioperative thirst is important, as they are also linked to the perception of the intensity and discomfort of thirst for the patient^(^ 16 ^,^ 20 ^)^.

The pre- and postoperative fasting time was the RF present in the entire sample studied. Fasting is part of the guidelines to ensure the safety of the anesthetic-surgical procedure, with a time of up to two hours being recommended for clear liquids^(^ 23 ^)^, with evidence showing improvement in the patient’s experience and reduction of discomforts, including thirst^(^ 9 ^)^. Nevertheless, it is identified that the implementation of adequate fasting times in clinical practice is challenging, even in institutions that aim to implement multimodal protocols^(^ 9 ^)^. The RF Dehydration, Insensible loss of hydration and Hypovolemia were observed in the IPP.

Dry mouth refers to dryness or lack of moisture in the oral cavity, caused by a decrease or absence of salivary flow. The reduction in salivary flow decreases the lubrication of the oral cavity, leading to the perception of roughness. It has a high prevalence in surgical patients, ranging from 69.2%^(^ 20 ^)^ to 87.3%^(^ 13 ^)^.

In cases of dryness of the labial mucosa, cracks, desquamation, inflammation, bleeding, whitish or reddish coloration and decreased mobility can be observed. This condition is reported by 22.5%^(^ 20 ^)^ to 79.1% of patients^(^ 13 ^)^.

Seven of the nine DCs remained in the model adjusted for the preoperative period. It was observed that the DC Dry throat, Dry lips, Thick saliva, Thick tongue, Constant swallowing of saliva, Desire to drink water showed better sensitivity adjustments. On the other hand, the DC Dry throat, Constant saliva swallowing and Caregiver’s report showed better specificity adjustment. The DC Dry throat and Constant saliva swallowing presented the best accuracy indicators in the model adjusted to the preoperative period. The prevalence of thirst was 62.6%, corroborating the high prevalence of thirst in this population^(^ 6 ^-^ 7 ^,^ 20 ^-^ 21 ^,^ 24 ^-^ 26 ^)^.

The DC Dry throat and Constant saliva swallowing presented better accuracy performance and were reported by patients with thirst in the perioperative period^(^ 25 ^-^ 27 ^)^. Swallowing saliva is one of the main strategies used by the patient in an attempt to relieve thirst, and can be performed through the administration of artificial components or mechanically or chemically stimulated by chewing gum^(^ 28 ^)^.

This finding corroborates research related to the neurophysiology of thirst and its relief. Swallowing movements (tongue protrusion, sensations from the tongue and swallowing) are linked to thirst relief, by anticipatory mechanisms related to pre-absorptive satiety^(^ 1 ^)^. The use of perioperative thirst relief strategies is based on this mechanism^(^ 2 ^,^ 21 ^,^ 29 ^-^ 31 ^)^.

The DC Bad taste in the mouth refers to an unpleasant taste and may be related to preoperative fasting time, or the use of medication during the anesthetic-surgical procedure. The sensation of a bad taste in the mouth is frequently reported by surgical patients and is associated with the presence of thirst^(^ 27 ^,^ 32 ^)^.

The dry mouth item was removed from the DC because, despite this characteristic being the most frequent in the studied sample, it is significantly linked to factors that cause thirst, assuming an etiological nature. Dry mouth is an important stimulus for fluid intake, which is caused by states of dehydration, medication actions or other causes^(^ 1 ^,^ 33 ^)^. Some hypotheses were raised by the authors as to why dry mouth did not present a good fit in the two models. A simple explanation is the sample size and characteristics. A larger sample in new studies may bring additional results that would broaden the discussion on this issue.

Physiological aspects and also aspects of understanding by the patient when they self-report their thirst need to be discussed in view of this result. The patient can interpret that dry mouth corresponds to the entire set of structures present in the oral cavity. The defining characteristics dry lips, constant swallowing of saliva and dry throat, thick saliva and thick tongue showed a frequency greater than 50% in the pre and IPP. These characteristics are related to the decrease in the humidity of the oral cavity and may overlap with the characteristic dry mouth in the identification by the patient.

Defining characteristics are observable indicators/inferences that are grouped together as manifestations of a diagnosis^(^ 14 ^)^. It is important to emphasize that not all clinical indicators are essential for attributing a diagnosis. Each of the indicators presents different gradients of commitment to the diagnosis and may be present or absent depending on the clinical spectrum of the diagnosis^(^ 34 ^)^.

Fasting is one of the main factors related to perioperative thirst. The mean number of fasting hours was 13:10 (±5:11) hours, a value higher than the time recommended by clinical evidence^(^ 23 ^)^. Excessive fasting time and its repercussions for the surgical patient are widely discussed in the medical literature, as well as the difficulty in adopting shorter times in clinical practice^(^ 5 ^,^ 9 ^,^ 19 ^,^ 35 ^)^.

Associated conditions refer to medical diagnoses, surgical procedures, prescription of pharmaceutical agents that are not modifiable independently by the professional nurse^(^ 14 ^)^. The AC Use of anticholinergics and Intubation are significantly associated with the presence of thirst, a fact explained by signs of dryness of structures in the oral cavity^(^ 1 ^,^ 7 ^,^ 19 ^,^ 35 ^)^.

A study that presented the risk factors for thirst, observed that the use of glycopyrrolate — an anticholinergic drug used to reduce saliva secretion — was the main risk factor for moderate to severe thirst in the IPP in the PACU (71.7% *versus* 66.4%, p = 0.047; adjusted odds ratio: 1.46, p = 0.013). This study demonstrates that anticholinergics are related to the presence and intensity of thirst in patients in the IPP^(^ 7 ^)^.

As a limitation of the study, the accuracy analyzes correspond to the population of a public teaching hospital in southern Brazil, restricting generalizations. New studies are suggested for comparison with the results presented in this research. We reiterate the relevance of investigating this diagnostic proposition in other scenarios that assist surgical patients, as well as pediatric surgical patients.

This study presents diagnostic accuracy values for the DC proposed by the perioperative thirst ND, increases the scientific evidence that supports the interpretations and judgment of this ND by nurses in the care of surgical patients.

## Conclusion

The study presents the validation of the proposition of a new nursing diagnosis called perioperative thirst. The analysis of the diagnostic components resulted in the finding that the DC Dry throat and Constant swallowing of saliva in the preoperative period and Bad taste in the mouth in the IPP presented the best measures of sensitivity and specificity, as well as the factors related to high ambient temperature and dry mouth showed a significant association with an increase in the probability of the patient being thirsty.

The determination of clinical indicators and etiological factors of thirst can help nurses to identify and carry out the correct management of perioperative thirst, extinguishing or reducing the discomfort and suffering in the surgical experience. Nurses have knowledge and effective tools to manage thirst in surgical patients, ensuring care with less discomfort.

The inclusion of this ND in the taxonomy expands care for surgical patients, strengthens the systematization of perioperative nursing care, highlights the autonomy of nurses in thirst management and expands clinical practice, teaching and research in this area.
